# Designing Nanoliposome-in-Natural Hydrogel Hybrid System for Controllable Release of Essential Oil in Gastrointestinal Tract: A Novel Vehicle

**DOI:** 10.3390/foods12112242

**Published:** 2023-06-01

**Authors:** Bulent Basyigit

**Affiliations:** Food Engineering Department, Engineering Faculty, Harran University, 63000 Sanliurfa, Turkey; bulentbasyigit@harran.edu.tr

**Keywords:** nanoliposome, maltodextrin, natural hydrogel, essential oil delivery, gastrointestinal tract

## Abstract

In this study, *thyme* essential oil (essential oil to total lipid: 14.23, 20, 25, and 33.33%)-burdened nanoliposomes with/without maltodextrin solution were infused with natural hydrogels fabricated using equal volumes (1:1, *v*/*v*) of pea protein (30%) and gum Arabic (1.5%) solutions. The production process of the solutions infused with gels was verified using FTIR spectroscopy. In comparison to the nanoliposome solution (NL_1_) containing soybean lecithin and essential oil, the addition of maltodextrin (molar ratio of lecithin to maltodextrin: 0.80, 0.40, and 0.20 for NL_2_, NL_3_, and NL_4_, respectively) to these solutions led to a remarkable shift in particle size (487.10–664.40 nm), negative zeta potential (23.50–38.30 mV), and encapsulation efficiency (56.25–67.62%) values. Distortions in the three-dimensional structure of the hydrogel (H2) constructed in the presence of free (uncoated) essential oil were obvious in the photographs when compared to the control (H1) consisting of a pea protein–gum Arabic matrix. Additionally, the incorporation of NL_1_ caused visible deformations in the gel (HNL_1_). Porous surfaces were dominant in H1 and the hydrogels (HNL_2_, HNL_3_, and HNL_4_) containing NL_2_, NL_3_, and NL_4_ in the SEM images. The most convenient values for functional behaviors were found in H1 and HNL_4_, followed by HNL_3_, HNL_2_, HNL_1_, and H2. This hierarchical order was also valid for mechanical properties. The prominent hydrogels in terms of essential oil delivery throughout the simulated gastrointestinal tract were HNL_2_, HNL_3_, and HNL_4_. To sum up, findings showed the necessity of mediators such as maltodextrin in the establishment of such systems.

## 1. Introduction

Many bioactive agents, including essential oils, have poor solubility, stability, and bio-accessibility. These deficiencies hamper their effective utilization in sectoral applications from the food to pharmaceutical areas [1]. Therefore, the scientific literature and related sectors have attempted to design novel possible strategies for unraveling these drawbacks. Among these strategies, one of the facile routes is the incorporation of bioactive agents into colloidal systems via the encapsulation technique. In line with this approach, liposomal modalities have recently been mounting curiosity in the encapsulation of natural products [2].

Liposomal systems are spherical self-assembly and cell-reassembly phospholipid vehicles formed by using an energy source in an aqueous periphery encircled by uni- and multi-amphiphilic lipid bilayer membranes [3,4]. They are constructed for a variety of applications, including wound healing [5], bioactive compound delivery [6], and so on. In addition, nanoliposomes are ideal carrier systems for delivering drugs, genes, or other therapeutic agents to specific targets in the body. For example, in a previous study, the effect of gemcitabine hydrochloride- and paclitaxel-loaded nanoliposomes on cancer cells were examined. The findings indicated that nanoliposomes limit/decrease cancer cell viability compared to free drugs [7]. Along with enhancing the therapeutic efficacy of drugs, nanoliposomes lead to an increase in pharmacokinetics and a reduction in toxicity in medical applications [8]. Additionally, the surface of nanoliposomes can be modified using ligands or antibodies to specifically target diseased cells or tissues, thereby enhancing the efficacy and reducing the side effects of the delivered drug [9,10]. In other words, the surface of nanoliposomes is modified by attaching specific ligands or antibodies that can recognize and bind to receptors or biomarkers present in the target cells. These ligands or antibodies are selected according to the characteristics of the desired organ or tissue. For example, if cancer cells in a particular organ are targeted, certain cell-specific antibodies are suffixed to the nanoliposome surface [11]. In this context, polyethylene glycol phospholipid-modified liposomes are present in the foreground. However, certain biological drawbacks regarding these systems have been reported. Therefore, there has been a trend in recent years towards the construction of lipid amphiphiles without the phospholipid component. Hydrophobic drug-anchoring sequence as an alternative to polyethylene glycol phospholipids is remarkable in this field [12]. In addition to these applications, the success of nanoliposomes in the encapsulation of hydrophobic and hydrophilic structures has been reported [13]. The versatile beneficial impact of liposomal systems on the essential oil encapsulation process has been highlighted elsewhere [14,15]. Protection from external forces [16], controlled release [17], and improved functionality (antioxidant and antimicrobial activity) are some examples of them [18]. In addition, the tendency of liposomal systems in the industry is associated with biocompatibility, non-toxicity, ubiquitous raw material in nature, and non-elusive/budget-friendly transactions [3,19]. On the other hand, the resilience of these systems to aggregation is poor and they have high deformability (low stability) when exposed to external stimuli and gastrointestinal fluids [4]. The establishment of liposome-in-hydrogel hybrid formulations to circumvent these undesirable characteristic properties that limit their usage is a promising strategy [20].

Hydrogels are three-dimensional polymeric networks consisting of interconnected hydrophilic chains. The unique characteristics of these systems are related to their remarkable water-absorbing properties (not dissolved in aqueous media) and their ability to swell tremendously without the destruction of their structural completeness [21]. They display versatile response attributes, namely, physical, chemical, and biological stimuli [22]. The building blocks of hydrogels are artificial polymers, natural polymers, or a combination of both. Acrylic acid, poly (N-isopropylacrylamide), poly (ethylene) glycol, and poly (vinyl alcohol) are examples of synthetic groups. Hydrogels fabricated in the presence of these polymeric structures possess advanced functional behavior and mechanical strength (strong gel integrity) compared to those fabricated naturally [23]. It is obvious that such features provide an advantage over artificial ones in diverse applications such as bioengineering, food, medicine, pharmaceuticals, personal care, and agriculture practices [1,24]. On the other hand, their toxic nature and non-environmental friendliness impose limitations on the utilization of artificial hydrogels [25]. Unlike artificial hydrogels, natural hydrogels have recently attracted considerable attention because of their cost-effectiveness, desirable biocompatibility, and biodegradability [26]. Polysaccharides (alginate, cellulose, chitosan) and proteins (whey protein, soy protein, pea protein) are decent materials for designing natural hydrogels [27,28,29]. These gel systems could be designed in different ways. For example, a previous study highlighted that the fabrication of alginate-based hydrogels was possible using an ionotropic gelation technique based on the interplay of anionic alginates with polyvalent inorganic cations [30]. In another study, an ion-exchange technique was used to design calcium alginate/cellulose nanocrystal hydrogels [31]. On the other hand, the usage of these materials alone could result in poor three-dimensional structures in gel systems. Therefore, one of the prerequisites for fabricating hydrogels with strength (desirable) networks is to incorporate them into the systems after the coexistence of polysaccharides and proteins. The modification of raw materials is another option for addressing related issues [25,32].

Tweens, spans, and cyclodextrins are included in the systems for the effective loading of the lipid-based (hydrophobic) platforms into hydrogels (hydrophilic medium) [33]. Cross-linked nanogel-coated liposome complexes were obtained using the Micheal addition reaction (cholesterol-loaded acryl groups were added to thiol groups) in a previous study [34]. In other words, liposomal formations could have an adverse effect on the three-dimensional network structure of hydrogels because of the poor harmony between them. In light of these considerations, the current study focused on the fabrication of pea protein–gum Arabic-based natural hydrogels containing thyme essential oil-loaded nanoliposomes added to maltodextrin solutions at various concentrations. An advanced spectroscopic tool was used to show the success of the nanoliposome production step and its mixing process with maltodextrin solutions, and the samples (nanoliposome and a mixture of nanoliposome and maltodextrin solutions) were characterized in terms of particle size, zeta potential, and encapsulation efficiency. The impact of the maltodextrin solution incorporation into nanoliposome systems on the morphological, functional, and mechanical properties of natural hydrogels was investigated. Additionally, the release behavior of essential oil from hybrid systems in the in vitro gastrointestinal tract was established.

## 2. Materials and Methods

### 2.1. Materials

*Thyme* essential oil was purchased from Arifoğlu Spice and Food Ind. Trade. Ltd. Co. (İstanbul, Turkey). Soybean lecithin was provided by Öz Yaldiz Chemical Industry Trade Ltd. Co. (İstanbul, Turkey). The purification process for soy lecithin was carried out according to a previous study [35]. Maltodextrin and gum Arabic were supplied from Sigma Aldrich Co. (St. Louis, MO, USA) and Merck (Darmstadt, Germany), respectively. Pea protein was obtained from Proteinocean Food Inc. (Ankara, Turkey). The remaining chemicals and solvents were procured from Sigma Aldrich and Merck unless otherwise stated.

### 2.2. Fabrication of Nanoliposome and Hydrogel Systems

#### 2.2.1. Fabrication Nanoliposomes Preparation

Nanoliposome systems were produced using the thin-film dispersion technique following the methods of Nahr et al. [36] with some modifications. Briefly, purified soybean lecithin and essential oil were entirely dissolved in chloroform (the amounts of soybean lecithin and essential oil are given in Table 1). The final mixture was kept at 4 °C to form a complex structure. After overnight incubation, the organic solvent was removed using a rotary evaporator (Buchi, Flawil, Switzerland) at 40 °C until a thin film was formed. Next, the film hydration step was performed with 2.5 mL of sodium phosphate buffer (10 mM, pH 7). These unstable systems with large particle sizes were subjected to the ultrasonication process by using Sonopuls UW2070 a high-speed high-shear ultrasonic homogenizer coupled with MS72 probes (Bandelin Electronic GmbH & Co. KG; Berlin, Germany). The conditions for this process conducted in an ice bath were 5 min, 5 cycles, and 50% power. Lastly, taking into account the amounts given in Table 1, the maltodextrin solutions were fully dissolved in 2.5 mL of sodium phosphate buffer (10 mM, pH 7) for 30 min at 150 rpm in a shaker and then slowly transferred to nanoliposome solutions (prior to incorporation to hydrogels) [37]. The related mixtures were freshly prepared prior to the analyses and hydrogel production.

#### 2.2.2. Hydrogel Preparation

The method described by He et al. [38] with some modifications was applied to the fabrication of natural hydrogel systems. Firstly, pea protein (30%, *w*/*v*) and gum Arabic (1.5%, *w*/*v*) solutions were stirred separately at 200 rpm for 5 h at room temperature (~24 °C) in a shaker (Lab-Line Instruments, Inc., Melrose Park, IL, USA). In order to ensure full hydration, the prepared solutions were kept at +4 °C overnight. Next, these stock solutions were combined in equal volumes (1:1, *v*/*v*) and the pH was shifted to 6.0 using a Hanna Instruments edge^®^ blue HI-2202 pH meter (Woonsocket, RI, USA). A total of 5 mL nanoliposome solution or a mixture of nanoliposome and maltodextrin solutions were added to a glass bottle containing 20 mL pea protein–carbohydrate blend, and a continuous mixing process was conducted for 1 h in a shaker at 200 rpm. The final mixture was transferred to a water bath (Nüve, Nüve ST 402, Ankara, Turkey) at 90 °C and held for 45 min. After equilibration to room temperature in an ice bath, the hydrogel systems were stored at +4 °C until testing time. Two control groups were constructed using the same method. The first was prepared without nanoliposomes or essential oil. In the other group, the essential oil not included in the nanoliposome (free essential oil) was incorporated directly into the hydrogels.

### 2.3. Analyses

#### 2.3.1. FTIR Spectroscopy

The solutions were dried using a Unopex B15 spray dryer (Unopex, İzmir, Turkey) with an air inlet temperature of 130 °C and a feeding rate of 5 mL/min [37].

The specific groups in soybean lecithin, maltodextrin, essential oil, and powders were examined using FTIR spectroscopy (IRTracer-100, Shimadzu Co., Kyoto, Japan). The resolution was set to 1/cm and spectrum scanning was performed between 600 and 4000 cm^−1^.

#### 2.3.2. Particle Size and Zeta Potential

The particle size and zeta potential measurements of samples were performed using a Zetasizer (Nano ZS90, Malvern Instruments, Worcestershire, UK) [39].

#### 2.3.3. Encapsulation Efficiency

Ethanol (50% *w*/*w*) was added to the tubes containing nanoliposome (the ratio of nanoliposome to ethanol was 1:7). This mixture was transferred to an amicon filter (Millipore Amicon Ultra-15) and centrifuged (Nüve NF 1200R, Nüve, Ankara, Turkey) at 4000 rpm for 5 min. The resulting filtrate was used for identifying free essential oil content. For total essential oil (free and encapsulated oil) content, samples were combined with chloroform via continuous mixing for 15 min. The organic phase was removed and used for measuring the total essential oil content. The absorbance of the filtrate and organic phase was read against a blank at λmax = 270 nm using a UV–vis spectrophotometer (Model UV-1700, Shimadzu Corp., Kyoto, Japan). For the blank, nanoliposomes without essential oil were mixed with chloroform for 15 min and the absorbance of the organic phase was measured. The following Equations (1) and (2) were utilized to compute the encapsulation efficiency [36,40]:

(1) $$Essential\;oil\left(\frac{mg}{mL}\right)=48.219\left({Abs}_{270}\right)-1.9634(R^{2}=0.99)$$

(2) $$Encapsulation\;Efficieny\left(\%\right)=\frac{encapsulated\;essentail\;oil\;content}{total\;essential\;oil\;content}\times 100$$

#### 2.3.4. Scanning Electron Microscopy

Freeze-dried hydrogels plated by gold–palladium under a vacuum were placed in the relevant part of the scanning electron microscopy (SEM) (ZEISS Sigma 300 Field Emission SEM, Oberkochen, Germany). The observation process was actualized at 1.00k× magnification [25].

#### 2.3.5. Water-Holding Capacity

The water-holding capacity of hydrogels was detected according to the method described by Wang et al. [41]. A small piece trimmed from filter paper was added to the tubes to retain the water separated from the samples before the hydrogels (~2 g) were centrifuged at 3000× *g* for 20 min. For the water-holding capacity computation, the following Equation (3) was employed:

(3) $$Water\;Holding\;Capacity\left(\%\right)=\frac{hydrogel\;mass\;after\;process}{initial\;hydrogel\;mass}\times 100$$

#### 2.3.6. Swelling Ratio

The cylindrical hydrogels (diameter: 8 mm × height: 10 mm) were transferred to a water bath at a temperature of 50 °C. After 30 min, the surface water was removed using a filter paper, and the swelling ratio was calculated according to the following Equation (4) [41]:

(4) $$Swelling\;Ratio\left(\%\right)=\frac{final\;hydrogel\;mass-initial\;hydrogel\;mass}{final\;hydrogel\;mass}\times 100$$

#### 2.3.7. Protein Leachability

Two grams of hydrogel were immersed in an 8 mL sodium phosphate buffer (0.05 M, pH 7) at room temperature in a glass beaker for 2 h. After centrifugation at 3000× *g* for 10 min, soluble (leached-out) protein in the supernatant was detected using the biuret method [42].

#### 2.3.8. Textural Behavior

The hardness, adhesiveness, and gumminess of the hydrogel systems were investigated using a TA-XT plus texture analyzer (Stable Micro Systems Ltd., Godalming, Surrey, UK) coupled with a cylindrical probe tip (P/0.5). The analysis was conducted at 5 °C using an HDP/90 platform [43].

#### 2.3.9. Rheological Behavior

The storage modulus (G′) and loss modulus (G″), which provide information about the viscoelastic behavior of hydrogels, were tested using the oscillation assay as a function of temperature (20–50 °C and at a heating rate of 5 °C/min). The samples were placed in the relevant part of the MARS II rheometer (Thermo Fisher Scientific, Karlsruhe, Germany), and measurements were actualized with parallel plate geometry (0.5 mm gap and 20 mm diameter plate) at a constant frequency (1.00 Hz) [25].

#### 2.3.10. In Vitro Gastrointestinal Release

The release behavior of essential oil was monitored under in vitro gastrointestinal simulation conditions (oral, gastric, and intestinal). Simulated saliva fluid (SSF), simulated gastric fluid (SGF), and simulated intestinal fluid (SIF) were prepared according to a method reported by a previous study [44].

For the oral phase, hydrogels were placed in a 4.8 mL simulated saliva electrolyte solution. Next, α-amylase (enzyme activity: 75 U/mL) and 25 µL of calcium chloride (0.3 M) were added to this mixture. After the pH was adjusted to 7.0 by adding sodium hydroxide (1 M), the total volume was adjusted to 9 mL with distilled water. The resulting mixture was exposed to agitation at 200 rpm for 2 min at 37 °C in a water bath.

For the gastric phase, 4.5 mL of oral bolus (saliva-digested hydrogel) was mixed with 4.095 mL of simulated gastric electrolyte solution. The digestion process was started at 200 rpm for 2 h at 37 °C in a water bath after the addition of pepsin (enzyme activity: 2000 U/mL), 2.25 µL calcium chloride (0.3 M), hydrochloric acid (1 M) (for setting up the pH to 3.0), and 402.75 µL of distilled water to this mixture.

For the intestinal phase, 4.1625 mL of the simulated intestinal electrolyte solution was added to a glass bottle containing 4.5 mL of chymus (gastric-digested hydrogel). The related solutions were combined with enzymes (trypsin activity: 100 U/mL; lipase activity: 2000 U/mL; α-amylase activity: 2000 U/mL), 45 mg of bile salts, 10 µL of calcium chloride (0.3 M), sodium hydroxide (1 M) (for setting up the pH to 7.0), and 328.50 µL of distilled water. Digestion was maintained at 200 rpm for 2 h at 37 °C in a water bath.

The centrifugation process at 5000 rpm for 15 min was applied to the digestive fluid at the end of each phase. The absorbance of the collected supernatant was measured at 270 nm, and the essential oil content was determined by using the equivalation given in Section 2.3.3 (Equation (1)). The release ratio of essential oil was detected using the following Equation (5):

(5) $$Release\;ratio\left(\%\right)=\frac{released\;essential\;oil\;after\;digestion}{initial\;essential\;oil}\times 100$$

#### 2.3.11. Statistical Analysis

All experiments including productions and analyses were performed at least in duplicate, and the collected datasets were presented as mean ± standard deviation. OriginPro 2021b (Origin Lab Inc., Northampton, MA, USA) was used for composing the graphs. Comparisons between means were performed using a one-way analysis of variance (ANOVA), followed by Tukey’s HSD test (*p* < 0.05). The related statistical results were obtained via the SPSS 22 statistical package for Windows (SPSS Inc., Chicago, IL, USA).

## 3. Results and Discussion

### 3.1. Authentication of Nanoliposome Systems

**Figure 1:**
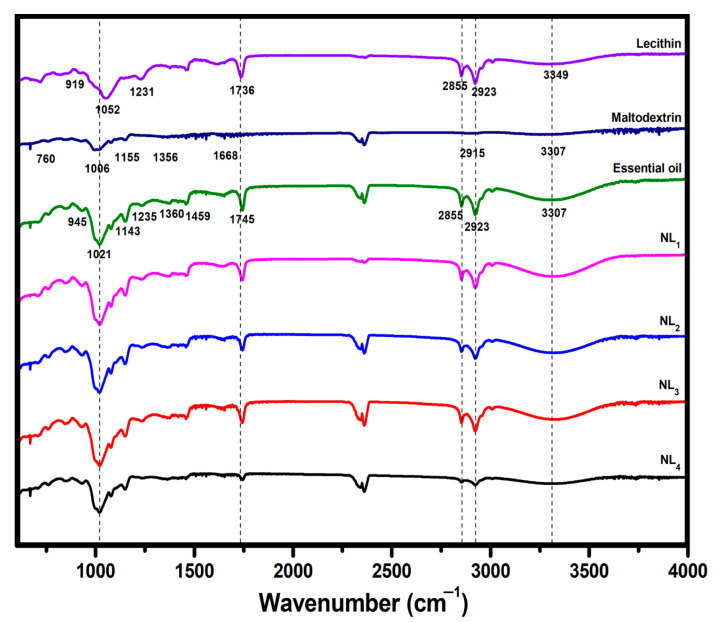
FTIR spectra of lecithin, maltodextrin, essential oil, nanoliposome, and nanoliposome–maltodextrin complexes. NL_1_ (lecithin: 0.20 M; essential oil to total oil (%): 14.23), NL_2_ (molar ratio of lecithin to maltodextrin: 0.80; essential oil to total oil (%): 20), NL_3_ (molar ratio of lecithin to maltodextrin: 0.40; essential oil to total oil (%): 25), and NL_4_ (molar ratio of lecithin to maltodextrin: 0.20; essential oil to total oil (%): 33.33).

FTIR analysis was conducted for the confirmation of nanoliposome and nanoliposome–maltodextrin complex production steps and spectra of the raw materials (lecithin, maltodextrin, and essential oil), and the final samples in the range 4000–600 cm^−1^ are illustrated in Figure 1. The spectra of all samples were predominated by the broad bands representing the stretching vibration of -OH groups (intra and inter-molecular hydrogen bonds) at a wavenumber of approximately 3330 cm^−1^. In other words, these bands represented alcoholic esters in lecithin and poly hydroxyl (phenols, alcohols, etc.) groups in the essential oil. The existing bands at 2923–2855 and 1736 cm^−1^ were responsible for the alkane group stretching (CH_2_) and the carbonyl group (C=O ester bond) vibration in lecithin, respectively. The other characteristic bands for this material were detected at 1231 cm^−1^ (P=O binding), 1052 cm^−1^ (C-N stretch in amino groups), and 919 cm^−1^ (phosphate ester: P-OR). Similar spectra have been reported for lecithin [36,45]. The visible absorption bands of maltodextrin at 2915, 1668, 1356, 1155, 1006, and 760 cm^−1^ verified the existence of the stretching vibration of the C-H bond of alkenes, carbonyl groups (C=O), aromatic ring vibrations, and stretching vibrations of the C-C bonds in the pyranose ring, respectively [46,47]. With respect to the essential oil, asymmetric stretching of C-H structures in aromatic compounds was identified at a wavenumber of 2923 cm^−1^. Symmetric stretching of these structures was identified at 2855 cm^−1^ [45]. Additionally, bands corresponding to the functional carbonyl group (1745 cm^−1^), -CH_3_ antisymmetric bond/-CH_2_ scissoring (1459 cm^−1^), C-H bending (1360 cm^−1^), and carboxyl acid/acetate stretching (1235 cm^−1^) in the essential oil appeared [15,36]. Together with the band at 1235 cm^−1^, the other three bands (1143, 1021, and 945 cm^−1^) indicated the presence of special structures (thymol and p-cymene) in the oil [40]. As for the final samples (nanoliposome and nanoliposome–maltodextrin complexes), bands related to the raw materials in the spectra of all systems were confirmed. Moreover, shifts were defined in their locations after the nanoliposome/nanoliposome–maltodextrin complexes production process, but their levels were negligible, indicating that this process was performed without considerable chemical interactions among lecithin, maltodextrin, and essential oil. Physical interactions have been reported for essential oil-loaded nanoliposome systems in previous findings [48].

### 3.2. Characteristic Attributes of Thyme Essential Oil-Loaded Nanoliposomes

The characteristic properties of the nanoliposome systems were defined by particle size, zeta potential, and encapsulation efficiency, and the results are presented in Table 2. The inclusion of maltodextrin into the systems displayed a remarkable influence on these related parameters (*p* < 0.05). The particle size of the nanoliposome (NL_1_) prepared in the absence of maltodextrin was 573 nm. This value dropped off with the incorporation of maltodextrin up to a certain point into the process and was measured as 521.90 nm for NL_2_ and 487.10 nm for NL_3_. However, particle size enhancement (664.40 nm) was observed with increasing maltodextrin amount from 400 mg (80 mg/mL) to 500 mg (100 mg/mL). This means that maltodextrin possesses a damaging impact on the stability of nanoliposome systems after a certain concentration [49]. This phenomenon was also supported by the zeta potential. In systems with desirable chemical and physical stabilities, the absolute value of the zeta potential is greater than 30 mV. In other words, the higher the value, the more stable is the colloidal suspension [50]. Electrophoretic mobility in the nanoliposomes prepared in the presence of 500 mg maltodextrin (NL_4_) was −29.10 mV, whereas this value was −38.30 mV for NL_3_. A similar trend was observed for encapsulation efficiency. The reduction in the particle size led to an increase in the encapsulation efficiency values. Smaller particles provide a larger surface area for the encapsulation material to interact with and encapsulate the target substance, resulting in enhancing encapsulation efficiency. In other words, smaller particles generally exhibit enhanced diffusion properties. This means that the encapsulation materials can more easily penetrate and distribute within smaller particles, leading to better encapsulation efficiency. This parameter showed an increasing trend with an increase in the maltodextrin concentration until a certain level (molar ratio of soybean lecithin to maltodextrin: 0.40). However, maintaining the insertion of maltodextrin into the vesicle systems was not a plausible approach in terms of encapsulation efficiency.

### 3.3. Semblance and Scanning Electron Microscopy Images of Hydrogels

**Figure 2:**
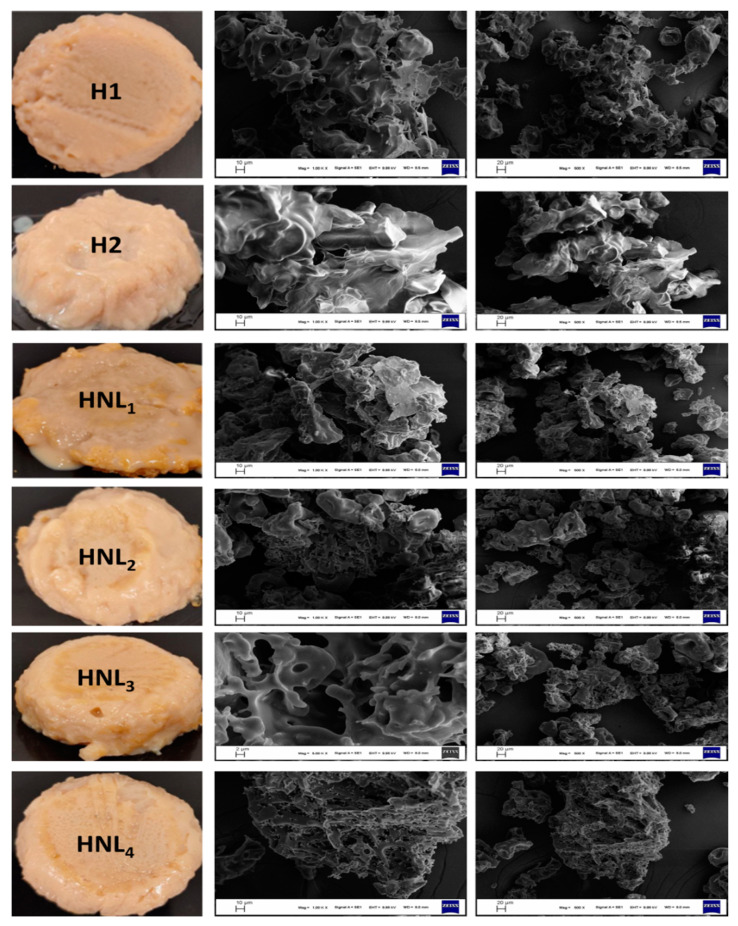
Appearances and SEM images of hydrogels. H1: Hydrogel without essential oil, nanoliposome, and nanoliposome–maltodextrin complexes. H2: Hydrogel containing essential oil alone. HNL_1_: Hydrogel containing NL_1_ lecithin: 0.20 M; essential oil to total oil (%): 14.23). HNL_2_: Hydrogel containing NL_2_ (molar ratio of lecithin to maltodextrin: 0.80; essential oil to total oil (%): 20). HNL_3_: Hydrogel containing NL_3_ (molar ratio of lecithin to maltodextrin: 0.40; essential oil to total oil (%): 25). HNL_4_: Hydrogel containing NL_4_ (molar ratio of lecithin to maltodextrin: 0.20; essential oil to total oil (%): 33.33).

Semblance and scanning electron microscopy (SEM) images of natural hydrogels are illustrated in Figure 2. Obviously, the composition of the nanoliposome systems exhibited a notable impact on modulating the three-dimensional structure of the hydrogels. This structure was destroyed with the addition of essential oil alone (H2) and nanoliposomes without maltodextrin (HNL_1_) into the gels compared to the control group (H1). However, the preparation of the hydrogels in the presence of nanoliposomes containing this carbohydrate was an effective way to substantially regain their lost structure. A fascinating (desirable) three-dimensional structure (similar to that of H1) was mainly observed in HNL_3_ and HNL_4_. This means that the number of temporary or reversible crosslinks between polymer chains through physical interactions, namely, hydrogen bonding, hydrophobic interactions, or electrostatic interactions, was higher in these hydrogels. The surface morphologies of the samples were in accordance with the photographs. The porous structures are characteristic of hydrogel systems [51]. The non-uniform pores were detected in H1, HNL_2_, HNL_3_, and HNL_4_, revealing that the presence of maltodextrin was beneficial for the three-dimensional structure. This beneficial behavior can be emanated from the compatibility between the hydrophilic systems and maltodextrin. Presumably, interactions between maltodextrin and the materials (pea protein and gum Arabic) used in the construction of hydrogels triggered the adaptation of nanoliposomes to hydrophilic conditions. Unlike H1, HNL_2_, HNL_3_, and HNL_4_, pores disappeared in H2 and HNL_1_, and intertwined sheet-like inhomogeneous/irregular and coarse structures became dominant. This phenomenon could be associated with the filling of lipid molecules into the pores [52]. Ultimately, incorporating essential oil-loaded nanoliposome/maltodextrin matrices into hydrogel systems was more reasonable to obtain the desirable three-dimensional structure and surface morphology, rather than directly adding essential oil or essential oil-loaded nanoliposomes. All the comments mentioned regarding the distinctions in the three-dimensional structure and morphology are supported by functional, textural, and rheological datasets.

### 3.4. Functional Behaviors of Hydrogels

The functional behaviors, namely, water-holding capacity, swelling ratio, and protein leachability of hydrogels, are outlined in this section and the results are presented in Table 3. The water-holding capacity was related to the ability of the gels to conserve the aqueous phase inside their network under centrifugal force (3000× *g*) [38]. The efficacy of the hydrogel nature on this ability was remarkable (*p* < 0.05) in the current study. The water-holding capacity of the hydrogel without essential oil, nanoliposome, and nanoliposome–maltodextrin complexes was 92.60%. The direct incorporation of essential oil and nanoliposome prepared in the absence of maltodextrin to hydrogels resulted in a decrease in this functional property, and the minimum water-holding capacity was detected in these hydrogels (H2: 68.03% and HNL_1_: 68.71%). The insoluble parts in the interior of the gels damaged the three-dimensional structure, which facilitated the separation of water molecules from them [53]. The presence of maltodextrin in the systems was capable of enhancing the hydrogels in terms of their related properties. Additionally, a positive correlation between the maltodextrin concentration and water-holding capacity was identified. The obtained values for the hydrogels containing nanoliposomes with maltodextrin were close to that of the control (H1), and the amount of immobilized water was highest in HNL_4_ (92.13%), followed by HNL_3_ (88.99%) and HNL_2_ (80.27%). Presumably, the contribution of maltodextrin could be associated with the bridge formed by the betwixt nanoliposomes and hydrogels due to its unique hydrophilic structure. In other words, maltodextrin suppressed the distortion of a three-dimensional structure by ensuring harmony between the hydrogel medium and lipid moieties. Additionally, the physical property, namely, pore size distribution, which provides easy penetration and retention of water within the gel network, could contribute to a greater water-holding capacity. These comments are in line with the photographs and textural behavior of the hydrogels. As shown in Figure 2 and Table 4, the hydrogels (H2 and HNL_1_) without this bridge possessed a poor network (weak three-dimensional structure). Ultimately, these structures were quite susceptible to disintegration and deterioration when centrifugation was applied. The existence of maltodextrin conferred rigid and strict blocks to gels, resulting in enhancing their ability to immobilize the aqueous phase [54]. Simply put, low stability against peripheral forces circumvents water trapping in the gel network [55].

The extra water-uptake capabilities of the gels represent the swelling ratio [56]. The findings regarding the swelling ratio of samples are shown in Table 3. The swelling ratio of H1 was 12.49%. A drastic decrease was observed in this value with the addition of free essential oil and maltodextrin-free nanoliposome into the hydrogels. The swelling ratios shifted to 4.65 and 4.84% for H2 and HNL_1_, respectively. Hydrophobic groups adversely affected the water-uptake ability of gels [57]. Moreover, the measurements revealed that the inclusion of maltodextrin into the system was a plausible way to enhance the swelling ability. The swelling ratio attained was 7.56 and 10.78% in HNL_2_ and HNL_3_, respectively. The water adsorption ability of the hydrogels culminated at the maximum concentration of maltodextrin (HNL_4_), which was measured as 13.96%. The results conflicted with certain physical properties of the gels. Hydrogels with a loose network structure exhibited superior swelling ability as more solvent molecules could accommodate within the polymer chains. Conversely, a dense network structure may limit this ability. Hydrogels with a looser network structure tend to have a higher swelling ratio because they can accommodate more solvent molecules between the polymer chains. Conversely, hydrogels with a dense network structure may have a lower swelling ratio [58]. This conflict could be ascribed to the partial dissolution of H2 and HNL_1_ with a looser network structure. In addition, the superior ability could result from the porous structures in H1, HNL_2_, HNL_3_, and HNL_4_. These structures may provide straightforward movement of water into the gel [59]. The results and comments supporting this approach are elaborated in the morphological observation section.

Protein leachability is the rate of protein transferred from the hydrogel systems to sodium phosphate buffer (0.05 M, pH 7.0). This rate is as low as possible in high-quality gels [42]. High protein leachability was detected in H2 (37.23%), followed by HNL_1_ (36.84%), HNL_2_ (25.51%), HNL_3_ (23.69%), HNL_4_ (11.12%), and H1 (10.90%). The leachability value close to H1 in the samples containing maltodextrin could be explained by a strong three-dimensional structure. When the gel images and mechanical properties were examined, the existence of free essential oil and maltodextrin-free nanoliposome solution with poor aqueous phase compatibility deteriorated the nature of the gel systems during gelation and did a disservice to the bonds between the protein and polysaccharide. Ultimately, the proteins were loosely attached to these systems and leached easily from the three-dimensional structures. In other words, as the proportion of maltodextrin solution increased (the lower the lecithin), the damage to the bonds between the pea protein and gum Arabic was minimized, and eventually, the proteins held on tighter within the system. This phenomenon could be due to the unique harmony between the aqueous phase and gum Arabic with maltodextrin. Naturally, the more maltodextrin, the better the compatibility of the matrices (essential oil loaded-nanoliposome/maltodextrin) to the systems, resulting in minimum protein leaching.

### 3.5. Textural Behaviors of Hydrogels

Textural attributes present a piece of preliminary information regarding the usage of gels in the food sector for pharmaceutical applications [60]. In this context, natural hydrogels have been characterized in terms of textural behaviors in this section, including hardness, adhesiveness, and gumminess, and the findings are presented in Table 4. The segregations in these parameters were obvious in the samples (*p* < 0.05). In comparison with the H1, the hardness value displayed a downward trend when free essential oil and maltodextrin-free nanoliposomes were directly incorporated into the gel systems. Moreover, minimal values were identified in these samples (H2 and HNL_1_). This means that H2 and HNL_1_ can easily explode when they are subjected to any process and/or storage compared to others [61]. The underlying reason for this phenomenon could be attributed to the attenuation of the inter-bond strength among the raw materials (protein and polysaccharide) utilized in gel construction because of the presence of lipid molecules. Insoluble moieties have the potential to damage the formation of the interpenetrating polymeric network in the gel systems [53]. Lower mechanical strength and softer textures in the semblance of H2 and HNL_1_ were visible. In return, the activation of maltodextrin led to the amelioration of the hardness value. This value increased gradually with an increase in the concentration of this polysaccharide and peaked in HNL_4_. Simply put, a possible explanation for this situation might be that maltodextrin facilitates the integration of nanoliposome solutions and hydrogel systems, leading to a tougher and denser network. As for the hardness, together with H1, the hydrogel systems with maltodextrin came to the forefront in terms of adhesiveness and gumminess. These systems exhibited less adhesive behavior compared to H2 and HNL_2_. A low adhesive value is an indication of quality [62]. In return, superior gumminess is evidence of stable gel systems [63]. Thus, the textural parameters were compatible with each other. Moreover, the desirable three-dimensional structure for hydrogel systems (especially HNL_4_) containing maltodextrin has also been emphasized elsewhere (photographs, SEM images, functional behavior, and rheological attributes) in the text. These aforementioned intercourses have been reported in a previous study [64].

### 3.6. Rheological Behaviors of Hydrogels

**Figure 3:**
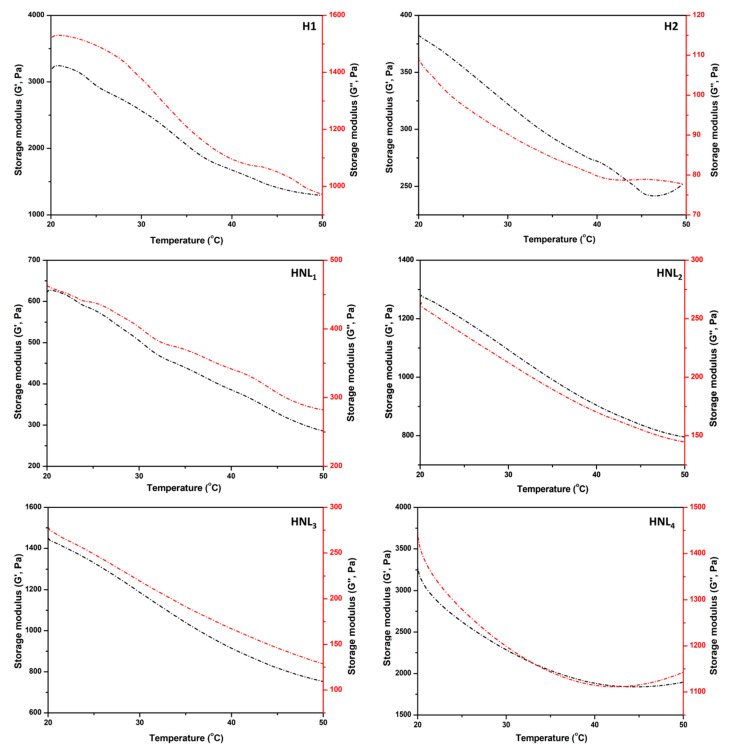
Rheological behaviors of hydrogels. H1: Hydrogel without essential oil, nanoliposome, and nanoliposome–maltodextrin complexes. H2: Hydrogel containing essential oil alone. HNL_1_: Hydrogel containing NL_1_ (lecithin: 0.20 M; essential oil to total oil (%): 14.23). HNL_2_: Hydrogel containing NL_2_ (molar ratio of lecithin to maltodextrin: 0.80; essential oil to total oil (%): 20). HNL_3_: Hydrogel containing NL_3_ (molar ratio of lecithin to maltodextrin: 0.40; essential oil to total oil (%): 25). HNL_4_: Hydrogel containing NL_4_ (molar ratio of lecithin to maltodextrin: 0.20; essential oil to total oil (%): 33.33).

The two parameters, namely, elastic modulus (G′) and viscous modulus (G″), used for interpreting the viscoelastic behavior of the hydrogels were assessed using an oscillatory rheometer as a function of temperature, and the curves are illustrated in Figure 3. Gels exhibited similar patterns and a continuous reduction was observed in these parameters when temperatures shifted from 20 to 50 °C. Increasing the temperature gave rise to a decline in the G′ and G″ values of the hydrogels [25,32]. In other words, gel (hard) and sol (soft) changeovers could occur, indicating that hydrogels may forfeit their initial stability with increasing temperature [65]. Moreover, the G′ values in all the gels remained above those of G″ during the measurements. This situation is evidence of the dominance of the elastic-like character rather than the viscous behavior in the samples [54]. Elasticity is one of the characteristic attributes of hydrogel systems [66]. On the other hand, the elasticity level is not independent of the nature of hydrogels [25,32]. Resembling the previous findings, the hierarchy in terms of elasticity was self-evident in the current study. The G′ value of H1 was more than five-fold higher than that of H2 and HNL_1_. These remarkable changes are evidence of transformation into a poor internal network. Lower G′ value in hydrogel systems is an indicator of structural imperfections [67]. This value exhibited an ascending trend in the systems composed of maltodextrin. The G′ value of the hydrogel containing the maximum concentration of this polysaccharide was quite similar to that of H1, revealing that integrating lipid-based materials into gel systems without notable damage to their internal structure is feasible. The related approach was proven not only in this section but also in other sections (photographs, SEM images, functional behavior, and textural properties) of the text. For example, crosslinking density representing superior elastic modulus and more solid-like behavior was distinguished in the physical appearance of hydrogels.

There is a strong relationship between the mechanical property of a product and its preference in the industry. In this context, their incorporation into hydrogels after pretreatment of nanoliposomes with polysaccharides such as maltodextrin could be a reasonable way to provide a desirable three-dimension and internal structure. Surely, this method will constitute a guide to various industries from food to pharmacy.

### 3.7. Release Behavior of Thyme Essential Oil during In Vitro Gastrointestinal Digestion

The released thyme essential oil ratio after sequential exposure of the hydrogel systems to the simulated oral, gastric, and intestinal fluids is tabulated in Table 5. The relationship between the leakage level of essential oil into the simulated fluids and the internal structure of the hydrogels was strong. The release to the medium within 2 min of the saliva stage was measurable in H2. This situation could be ascribed to the starting of a partial burst in its structure because of a weak network structure. In other words, the hydrogel matrix, whose three-dimensional structure was not at the desired level, gradually eroded, leading to a faster release. The essential oil residues on the gel surface may be another factor. In return, other gel systems maintained their intactness after exposure to saliva digestion, and no release was detected. This phenomenon can be explained by three factors. One of them may have been the short process time (2 min). The second explanation could be the absence of enzymes affecting the proteins forming the main structure of the gel systems and lipid-based vehicles. The superior mechanical properties may be another comment compared to H2. No notable release occurred during saliva secretion [68]. The release of essential oil was slightly accelerated when it passed through the gastric phase. The maximum transition from hydrogels to gastric fluid was detected in H2 (21.81%), followed by HNL_4_ (10.42%), HNL_3_ (9.58%), HNL_2_ (9.35%), and HNL_1_ (8.29%). The lower protective behaviors of a hydrogel containing free essential oil could be attributed to the disintegration of proteins (the building blocks of hydrogels). A similar situation was also valid for HLN_1_, HNL_2_, HNL_3_, and HNL_4_. However, the essential oil in these gel systems was loaded into lipid-based vehicles that were not digested in the gastric phase. In other words, these conditions were not convenient for the degradation of lipids. Ultimately, the inhibition in terms of the release was greater in hydrogels containing nanoliposome-embedded essential oil. Moreover, preparing nanoliposomes with higher lecithin concentrations was a way for prolonging the essential oil release under simulated gastric conditions. As expected, the release became faster after immersion in intestinal fluid. Resembling the gastric phase, the leak rate in the related step was the highest in H2 (45.92%). This could be attributed to structural drawbacks and the continued dismemberment of protein/polysaccharides because of enzymatic (trypsin and α-amylase) action. On the other hand, the hierarchy was altered in the intestinal phase, and the second largest release rate was found in HNL_1_ (32.97%). Bursting of this hydrogel due to its poor network structure could lead to nanoliposomes containing essential oil coming into direct contact with the enzyme (trypsin) in the intestinal phase (initiation of the lipid breakdown), indicating that vehicles may be demolished easily and eventually to increase the diffusion rate. A similar situation was not observed in HNL_2_, HNL_3_, and HNL_4_ due to their sturdy (tougher and denser) three-dimensional structures, and the amount of essential oil that passed to the intestinal fluid was lower in these hydrogels compared to HNL_1_. In addition, the non-negligible release rate in HNL_2_, HNL_3_, and HNL_4_ can be attributed to their characteristic pore structures. The intestinal fluid may penetrate the interior of the gels by using these pores, leading to erosion [69]. To sum up, these findings obviously indicate that such systems could be promising candidates for the delivery of bioactive compounds (phenolic antioxidants, drugs, etc.) in different applications, especially in all areas of the healthcare industry.

## 4. Conclusions

This study presented momentous datasets regarding the coexistence of hydrophilic and hydrophobic systems. Moreover, the contribution of these findings to the scientific literature is undeniable in terms of the usage of non-toxic and biodegradable hydrogels in the encapsulation of lipid-based materials. The direct incorporation of essential oil and nanoliposomes has led to a great mechanical detriment in the hydrogel systems. Therefore, the addition of nanoliposome solutions containing essential oil prepared in the presence of polysaccharide (maltodextrin) to these systems was a reasonable approach to providing three-dimensional network structures. More pronounced findings in terms of appearance and porous structure were detected in HNL_4_. These acceptable network structures increased the surface area of the HNL_4_ hydrogel after treatment, thus enabling it to exhibit superior functional properties (i.e., water-holding capacity, swelling rate, and protein leaching). Additionally, the same hydrogel samples were more applicable in terms of mechanical properties, according to the temperature sweep and textural analysis results. Moreover, they are promising systems for enhancing the stability of essential oil under simulated gastrointestinal conditions. In other words, the gel systems with desirable internal networks are suitable for the controlled diffusion of essential oil loaded into nanoliposomes. This behavior demonstrates their potential for the delivery of bioactive compounds, revealing that they can be integrated into many fields from engineering (food packing material and tissue engineering) to medical applications (drug delivery). Further studies concerning hydrogels containing essential oil-loaded nanoliposomes are indispensable to affirm these assumptions in these related fields.

## Figures and Tables

**Table 1:** Experimental conditions for the mixture of nanoliposome and maltodextrin solutions.

Code	Molar Ratio of Soybean Lecithin to Maltodextrin	*Thyme* Essential Oil to Total Lipid (%)
NL_1_	0.20 M/- *	14.23
NL_2_	0.80	20
NL_3_	0.40	25
NL_4_	0.20	33.33

* Without maltodextrin.

**Table 2:** Size, zeta potential, and encapsulation efficiency of thyme essential oil-loaded nanoliposomes with/without maltodextrin solution.

Code	Particle Size (nm)	Zeta Potential (mV)	Encapsulation Efficiency (%)
NL_1_	573.00 ± 4.04 ^b^	−23.50 ± 0.17 ^c^	56.25 ± 0.92 ^d^
NL_2_	521.90 ± 5.72 ^c^	−28.40 ± 2.11 ^b^	60.39 ± 1.75 ^c^
NL_3_	487.10 ± 7.41 ^d^	−38.30 ± 1.06 ^a^	67.62 ± 0.64 ^a^
NL_4_	664.40 ± 2.95 ^a^	−29.10 ± 1.26 ^b^	63.92 ± 0.56 ^b^

Data are given as mean ± standard deviation. Discrete lowercase letters (^a–d^) in the same row are used to explain statistical differences (*p* < 0.05). NL_1_ (lecithin: 0.20 M; essential oil to total oil (%): 14.23), NL_2_ (molar ratio of lecithin to maltodextrin: 0.80; essential oil to total oil (%): 20), NL_3_ (molar ratio of lecithin to maltodextrin: 0.40; essential oil to total oil (%): 25), and NL_4_ (molar ratio of lecithin to maltodextrin: 0.20; essential oil to total oil (%): 33.33).

**Table 3:** Water-holding capacity, swelling ratio, and protein leachability of hydrogels.

Code	Water-Holding Capacity (%)	Swelling Ratio (%)	Protein Leachability (%)
H1	92.60 ± 0.72 ^a^	12.49 ± 0.43 ^b^	10.90 ± 0.07 ^e^
H2	68.03 ± 0.07 ^d^	4.65 ± 0.07 ^e^	37.23 ± 0.48 ^a^
HNL_1_	68.71 ± 0.97 ^d^	4.84 ± 0.76 ^e^	36.84 ± 0.10 ^a^
HNL_2_	80.27 ± 0.22 ^c^	7.56 ± 0.16 ^d^	25.51 ± 0.17 ^b^
HNL_3_	88.99 ± 0.61 ^b^	10.78 ± 0.54 ^c^	23.69 ± 0.07 ^c^
HNL_4_	92.13 ± 0.82 ^a^	13.96 ± 0.37 ^a^	11.12 ± 0.03 ^d^

Data are given as mean ± standard deviation. Discrete lowercase letters (^a–e^) in the same row are used to explain statistical differences (*p* < 0.05). H1: Hydrogel without essential oil, nanoliposome, and nanoliposome–maltodextrin complexes. H2: Hydrogel containing essential oil alone. HNL_1_: Hydrogel containing NL_1_ lecithin: 0.20 M; essential oil to total oil (%): 14.23). HNL_2_: Hydrogel containing NL_2_ (molar ratio of lecithin to maltodextrin: 0.80; essential oil to total oil (%): 20). HNL_3_: Hydrogel containing NL_3_ (molar ratio of lecithin to maltodextrin: 0.40; essential oil to total oil (%): 25). HNL_4_: Hydrogel containing NL_4_ (molar ratio of lecithin to maltodextrin: 0.20; essential oil to total oil (%): 33.33).

**Table 4:** Hardness, adhesiveness, gumminess, and resilience values of hydrogels.

Code	Hardness	Adhesiveness	Gumminess
H1	522.83 ± 2.34 ^a^	−87.59 ± 1.47 ^b^	92.31 ± 01.33 ^a^
H2	60.31 ± 1.43 ^e^	−23.53 ± 1.05 ^c^	17.71 ± 0.60 ^e^
HNL_1_	123.01 ± 4.97 ^d^	−22.26 ± 1.23 ^c^	38.81 ± 1.29 ^c^
HNL_2_	288.19 ± 2.46 ^c^	−97.77 ± 1.12 ^a^	34.46 ± 0.89 ^d^
HNL_3_	380.87 ± 1.64 ^b^	−86.94 ± 2.26 ^b^	50.01 ± 2.15 ^b^
HNL_4_	522.01 ± 3.79 ^a^	−87.45 ± 1.35 ^b^	91.02 ± 3.16 ^a^

Data are given as mean ± standard deviation. Discrete lowercase letters (^a–e^) in the same row are used to explain statistical differences (*p* < 0.05). H1: Hydrogel without essential oil, nanoliposome, and nanoliposome–maltodextrin complexes. H2: Hydrogel containing essential oil alone. HNL_1_: Hydrogel containing NL_1_ lecithin: 0.20 M; essential oil to total oil (%): 14.23). HNL_2_: Hydrogel containing NL_2_ (molar ratio of lecithin to maltodextrin: 0.80; essential oil to total oil (%): 20). HNL_3_: Hydrogel containing NL_3_ (molar ratio of lecithin to maltodextrin: 0.40; essential oil to total oil (%): 25). HNL_4_: Hydrogel containing NL_4_ (molar ratio of lecithin to maltodextrin: 0.20; essential oil to total oil (%): 33.33).

**Table 5:** The release ratio of essential oil in simulated gastrointestinal fluid.

Code	SSF (%)	SGF (%)	SIF (%)
H2	1.86 ± 0.12	21.81 ± 0.96 ^a^	45.92 ± 1.18 ^a^
HNL_1_	nd	8.29 ± 0.12 ^d^	32.97 ± 0.72 ^b^
HNL_2_	nd	9.35 ± 0.21 ^c^	20.64 ± 0.57 ^d^
HNL_3_	nd	9.58 ± 0.37 ^c^	20.35 ± 0.41 ^d^
HNL_4_	nd	10.42 ± 0.16 ^b^	23.87 ± 0.22 ^c^

Data are given as mean ± standard deviation. Discrete lowercase letters (^a–d^) in the same row are used to explain statistical differences (*p* < 0.05). SSF: Simulated saliva fluid. SGF: Simulated gastric fluid. SIF: Simulated intestinal fluid. H2: Hydrogel containing essential oil alone. HNL_1_: Hydrogel containing NL_1_ lecithin: 0.20 M; essential oil to total oil (%): 14.23). HNL_2_: Hydrogel containing NL_2_ (molar ratio of lecithin to maltodextrin: 0.80; essential oil to total oil (%): 20). HNL_3_: Hydrogel containing NL_3_ (molar ratio of lecithin to maltodextrin: 0.40; essential oil to total oil (%): 25). HNL_4_: Hydrogel containing NL_4_ (molar ratio of lecithin to maltodextrin: 0.20; essential oil to total oil (%): 33.33). nd: not detected.

## Data Availability

The data presented in this study are available on request from the corresponding author.
